# RETRACTION: Rhubarb Tannins Extract Inhibits the Expression of Aquaporins 2 and 3 in Magnesium Sulphate-Induced Diarrhoea Model

**DOI:** 10.1155/bmri/9768597

**Published:** 2025-07-31

**Authors:** BioMed Research International

RETRACTION: C. Liu, Y. Zheng, W. Xu, H. Wang, and N. Lin, “Rhubarb Tannins Extract Inhibits the Expression of Aquaporins 2 and 3 in Magnesium Sulphate-Induced Diarrhoea Model,” *BioMed Research International* 2014 (2014): 619465, https://doi.org/10.1155/2014/619465.

The above article, published online on 18 August 2014 in Wiley Online Library (http://wileyonlinelibrary.com), has been retracted by agreement between the Editorial Board and John Wiley & Sons Ltd. The retraction has been agreed following the investigation of figure concerns initially raised on PubPeer [1]. Specifically:

• In Figure 4a, GADPH lanes 2-5, Figure 6d GAPDH lanes 1-4 and Figure 7a GADPH lanes 2-5 are identical.
• Figure 4b GADPH lanes and Figure 6b GADPH lanes are identical. Additionally, Figure 4b GADPH lanes are identical to Figure 5a and 6a GADPH lanes in [2]
• Figure 6a GADPH lanes and Figure 7b GADPH lanes are identical.

Additionally, the authors reported that the Figure 3a AQP2 negative control, Figure 3b AQP3 negative control, Figure 5a AQP2 negative control and Figure 5b AQP3 negative control are incorrect. The corresponding DAPI images were used by mistake during image preparation.

The authors explained that the errors were made during image preparation; however, their response did not satisfy our concerns and it is therefore retracted.

The authors did not respond when asked to approve the retraction and its notice.
